# Allergy and Aging: An Old/New Emerging Health Issue

**DOI:** 10.14336/AD.2016.0831

**Published:** 2017-04-01

**Authors:** Massimo De Martinis, Maria Maddalena Sirufo, Lia Ginaldi

**Affiliations:** Department of Life, Health, & Environmental Sciences, University of L'Aquila, L'Aquila, Italy

**Keywords:** aging, allergy, elderly, immunosenescence, translational immunology, geriatrics

## Abstract

Allergy reactions are the most common immunological diseases and represent one of the most widespread and fast growing chronic human health problems among people over 15 years of age in developed countries. As populations get older worldwide, allergy manifestations in aged persons will occur more often in the future. To date, there has been much more studies on allergies in children than in adults. As the population ages, clinicians must be prepared to meet all the elderly's health care needs, including these new and emerging health issue. Allergic diseases represent an old/new emerging health issue. Because many common illnesses masquerade as atopic disease, the differential diagnosis of suspected allergic diseases becomes more expanded in an aging population. Research in the field needs to focus on both human and animal model systems to investigate the impact of the aging process on the immunologic pathways underpinning allergy and its different facets.

## Background

In industrialized countries the proportion of elderly people is expanding and in many of them especially the group of persons 65 years or older is the most rapidly expanding subpopulation, and the proportion of persons older than 80 years will even rise in a disproportionate manner. By 2040, people aged >65 years will represent about 25% of the people in industrialized countries, and the proportion of persons older than 80 years will rise in a disproportionate manner. Even if life expectancy is greater today than in the past, there are no major changes in the spectrum of diseases affecting old age. Therefore, issue of the health of the elderly has become a topic in modern medicine [1].

Immediate hypersensitivity (type I) is the most common immunological disease and represents the most widespread and fast growing chronic human health condition in people aged 15th years and older.

About 25% of the population is affected by type I reactions in industrialized countries, with manifestations which range from severe life-threatening to irritating conditions [2]. Environmental changes of the last decades such as climate, pollution, dietetic, and items such as mode of delivery (increased use of caesarean section) that shapes microbial colonization patterns, could be among the causes of the fast growth of allergy and other non communicable diseases prevalence in a relatively short period of time. A chronic low inflammatory state due to all these factors trigger and maintain the allergic and other inflammatory conditions [3].

Although a steadily increase of the aging population is observed, yet little research has been conducted to assess the exact prevalence of allergic diseases and atopy in the elderly. Allergic disorders will become always more frequent in the future as people in the world get older (Table 1). To date, most studies on allergy prevalences were conducted in childhood than in adulthood. Some age-related decline of physiological functions like gastric pH elevation and vitamin D fall, may induce symptoms capable of masking allergic diseases in aged people and this may represent a big challenge to epidemiology [4].

**Table 1:** Allergy in the elderly: a panoramic view

• The elderly population is steadily increasing and issues related to his state of health will become a topic of increasing relevance. • With increasing age, numerous underlying physiological changes occur, and the risk of chronic diseases rises. At the same time allergy is increasing worldwide, and 5-10% of allergies is affecting elderly people. • Taking into consideration the dramatic increase of all forms of allergies during the last decades also in the elderly, they are considered a real "epidemic" of the XXIst century, being classified by the WHO as the fourth most frequent chronic diseases. • Despite the great importance of the problem, allergic diseases, compared to all other chronic diseases, are neglected and undervalued. • In old people a number of factors may contribute to trigger allergy and/or to disguise it, making it longer and difficult to achieve the right diagnosis and relief suffering persons often already affected by several other diseases and in presence of many other causes of frailty. • The multifaceted dynamics among multimorbidity, disease, and underlying physiological change, can result in health states in older age that are not captured by traditional disease classifications and not easily diagnosed. • Many epidemiological surveys have shown that the number of allergic patients in Europe and other developed and developing countries is increasing dramatically. A notable proportion of individuals with respiratory allergy in Europe are underdiagnosed, undertreated and dissatisfied with their treatment and among these most are elderly people. • Recently there has been a large increase in knowledge about the immunological processes that play a role in allergic diseases and about environmental exposure (irritants and allergens), a tendency that will continue, and this gain in knowledge has led to changes in diagnostic and therapeutic possibilities (component-resolved diagnosis, new forms of immunotherapy), and to a better understanding of the role and the possibilities of primary and secondary prevention (benefits and risks of allergen avoidance, infant feeding, application of pro-/prebiotics, risk of tobacco smoke, role of epigenetics).

In addition to functional and structural changes observed in the elderly, a major topic of discussion is the relevance of the effects of drug therapies and/or immunological modifications on allergic manifestations in aged people. In the next future, the issue of allergy in adults and particularly in the elderly will be of great and great importance (Table 2) [5].

The prevalence of allergy in older people was reported by Milgrom et al. from 5 to 10% and appears to increase [6] while significantly higher data came from the reports of Wüthrich et. al: prevalence rates of atopy were 36.4% in men aged aged from 18 up to 60 years versus 26.2% in men aged >60 years and 30.6 and 18.1% in women, respectively; prevalence rates of self-reported allergic rhinitis in subjects >60 years old were 13.0% for men and 15.4% for women, and for doctor's diagnosed asthma 6.6% versus 7.6%, respectively [7]; and from the study by Bozek et al.: atopy was diagnosed in 26.7% of patients (26.7% of women and 26.6% of men). The average morbidity associated with age and sex in this population was 5.9% for bronchial asthma, 1.6% for atopic dermatitis/eczema, 12.6% for seasonal allergic rhinitis, 17.1% for perennial allergic rhinitis, and 6.4% for combination of the respective allergic diseases [8].

Two aspects occur in parallel in the onset of allergic diseases during aging: the individual, genetic and epigenetic influenced, immunosenescence, and the external "risk factors" including, for example, internal diseases; their respective interference might affect the development and the type of allergic reactions. Immunosenescence refers to changes of immune function observed with aging and tissue structure modifications typical of advanced age [9,10]. Concurrent diseases, polydrug therapy and adverse drug reactions could be frequent additional risk factors. A full immune response against new antigens is more difficult and slow in old people and could be not completely protective. A compromission of the integrity of epithelial barriers, a subclinical, chronic inflammatory condition and an enhanced Th2 (allergic) immune response play central roles in driving the onset of allergies [6].

## Immunosenescence

The aging process impacts on body functions at the molecular, cellular and systemic levels, leading to loss of homeostasis, decreased ability to respond to extrinsic and intrinsic challenges, and increased susceptibility to chronic inflammatory diseases, resulting in senescence. Moreover, micronutrients with important immunoregulatory roles, such as iron, zinc and vitamin D, are often deficient in the elderly [11,12]. Aging mainly reflects the consequence of lifelong unrepaired damages, and is characterized by a complex phenotype associated with progressive changes in many organs and systems [13].

**Table 2:** key issues

• The issue of the health of the elderly has become a topic in modern medicine as older people represent the most rapidly expanding part of the population and reached the highest levels in the history • Immediate hypersensitivity (type I) is the most common immunological disease. It represents the most widespread and fast growing chronic human health condition in people aged 15th years and older in industrialized countries. About 25% of the population is affected, with manifestations which range from only irritating to severe life-threatening conditions • Allergic diseases are mostly caused by inhalant allergens, in particular mold and pollens and climate changes modify their deployment and amount • The onset of allergic diseases in older people is driven by immunosenescence, the changes of immune function observed with aging and tissue structure modifications typical of advanced age. Concurrent diseases, drug polytherapy and adverse drug reactions could be frequent complications • The contribution of epigenetic regulation to allergic diseases is a crucial topic. Discovering and validating epigenetic biomarkers linking exposure to allergic diseases could help to a better definition of risk, prognosis, response to therapy and development of novel treatments • Collaborative translational and interdisciplinary research is needed to identify new biomarkers of disease, accounting for the unique phenotypic makeup of the elderly allergic and for the immunological and physiological changes associated with the natural process of aging that affect diagnosis and management

In particular, the immune system is deeply remodeled during aging. Several mechanisms of both innate and adaptive immunity undergo age-related changes, configuring the so-called immunosenescence. Many aspects of immune function decline with aging, while others become more active. The main hallmarks of immunosenescence are lymphocyte subpopulation imbalances (decreased naïve and increased memory lymphocytes with accumulation of dysfunctional senescent cells with shortened telomeres), thymus involution with decreased new T cell generation, hematopoietic stem cell dysfunctions [14], defects in apoptotic cell death, mitochondrial function and stress responses, malfunctioning of immune regulatory cells. As a consequence, a senescent immune system is characterized by impaired interactions between innate and adaptive immune responses, continuous reshaping and shrinkage of the immune repertoire by persistent antigenic challenges, and chronic low-grade inflammation [15,16]. These changes lead to an increased susceptibility to newly encountered infections as well as to a shift of the immune system towards an inflammatory, autoimmune, Th2 profile. This immune dysregulation provides the background for the development of the increased susceptibility to infections and autoimmune diseases, neoplasias, metabolic diseases, osteoporosis, neurological disorders, as well as to allergic inflammation [11].

Therefore, it is evident that the whole immune system is heavily impacted by aging. Classical immunological defence functions, such as the defence against infections and neoplasia, as well as inflammation and allergic reactions, are modified in the elderly. The prevalence and presentation of both allergies and classical immune mediated diseases, mainly autoimmunity and immunodeficiency, are also peculiar in the elderly. Moreover, the age-related modified interactions between the different components of the immune system (cellular and humoral immunity, innate and specific immune functions, ecc.) determines a complicated immunological profile in the elderly that facilitates the development of chronic inflammatory reactions and allergies.

## Brain, aging and the stress response

Age-related changes occur in all parts of the body, including the brain. Damage by free radicals and inflammation increase and changes in neurotransmitters affect communication between neurons. Neuro-degenerative diseases, cognitive impairment, depression and poor compliance to stressors are among the major neurological findings in the elderly.

Stress releases hormones and other molecules, including histamine, leading to allergy symptoms. While stress is not actually cause of allergies, it can make an allergic reaction worse by increasing histamine release. Therefore, stress and allergies mutually reinforcing. Aging is considered the consequence of the lifelong accumulation of the effects of stressors such as physical (UV), metabolic (ROS) and immunological (antigens) agents, which the body becomes unable to counteract, leading to tissue damage, chronic inflammation and possibly occurrence of allergies [17].

Psychological stress is recognized at the onset or aggravation of multiple allergic diseases. Inflammatory mediators are likely involved. For example, skin, as well as gut and lung, is not only target of key stress mediators, but also a local source for factors inducing various immune and inflammation responses. Stress conditions exert their effects mainly through the hypothalamic-pituitary-adrenal axis and through ACTH and glucocorticoids. Peripheral nerves can also impact tissue health through secreted factors like neuropeptides (substance P or SP) and neurotrophins. They serve as local stress responders that mediate neurogenic inflammation facilitating allergic inflammation. Mast cells, in particular skin mast cells, are central player of the stress responses and neurogenic inflammation. Moreover, stress can affect various aspects of mast cell functions, including survival, activation, and downstream secretion of various vasodilatory and proinflammatory mediators, such as histamine, VEGF, cytokines, nitric oxide, and proteases. These mediators are also involved in eliciting allergic reactions. All major stress pathways are affected by aging [18].

**Figure 1.**
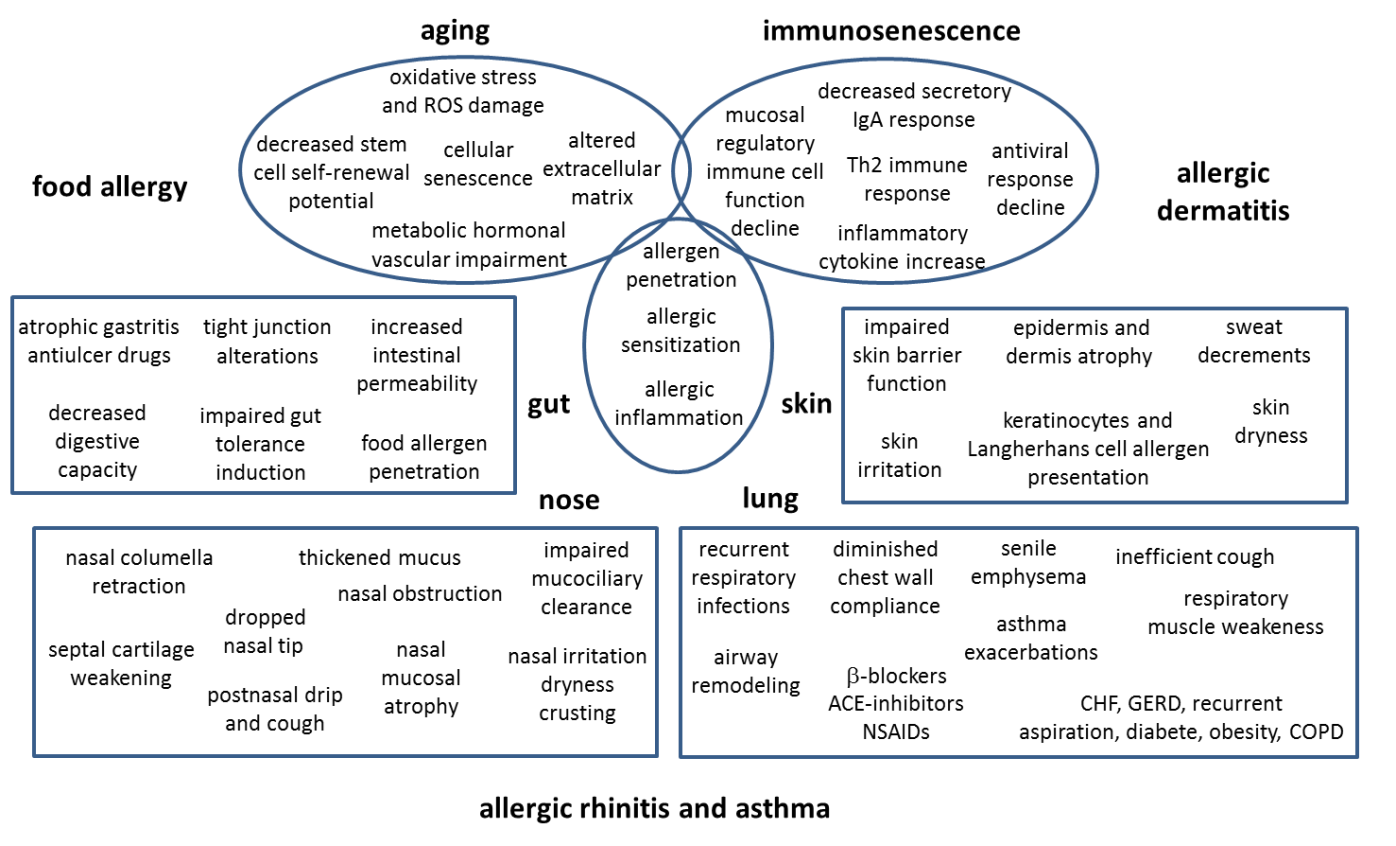
**Age-related changes underlying allergy in the elderly**. The onset of allergic diseases in the elderly is driven by cell aging at large and by immunosenescence and tissue structure modifications typical of advanced age. The figure illustrates the age-related functional and structural changes in upper and lower airways, skin and gut underlying the onset of allergy reactions in the elderly. ACE = angiotensin-converting-enzyme; CHF = chronic heart failure; COPD = chronic obstructive pulmonary disease; GERD = gastroesophageal reflux disease; NSAIDs = nonsteroidal anti-inflammatory drug; PPI = proton pump inhibitors.

## Pharmacokinetics and pharmacodynamics in the elderly

Aging does not diminish the absorption of most medications that by diffusion permeate the gastrointestinal epithelium, nevertheless the increase in total body fat and the reduction in lean body mass cause an altered distribution of the drug. The volume of distribution changes between individuals because of different protein-binding capacity of the body, total body water and amount of lean and adipose tissue. In elderly women the proportion of adipose tissue relative to total body weight increases 33% to 48%, while in elderly man 18% to 33%, respectively. As becoming older, total body water decreases by as much as 15%, both extracellular and intracellular. People assuming long-term treatment with diuretics may show even higher loss of extracellular water. Coronary, brain and skeletal muscles perfusion is maintained at nearly normal levels in the elderly, while it decreases in the gastrointestinal tract, liver and kidneys, and the cardiac output decreases. The drug action duration and the drug half-life both depend on the volume of distribution. Liver drug metabolism in the elderly continues to represent a discussed topic despite extensive research. At last, all drugs exhibit a reduced metabolism in older people while cytochrome P-450 substrates show a longer half-life. In the elderly we must consider the pharmacodynamics of a drug in addition to the pharmacokinetics.

Homeostatic mechanisms may change with aging, as well as, we can observe alterations at signal-transduction or receptor level. A gradual reduction of homeostatic mechanisms represents one of the key changes that characterize the aging process. The reduced counterregulatory measures typical for the elderly can be explained because more time is required to regain the original steady state and a less attenuation of drug effects occurs with aging.

The WHO defines any unintended, undesired, noxious drug effect occurring at doses used for treatment, diagnosis or prevention as an “adverse drug reaction”. Whenever drugs are prescribed for older people, several clinical and medication induced risk factors should be considered in addition to the already described numerous physiopathological changes due to the aging process itself. A progressive reduction of stimuli induced adaptation capabilities accompany aging. In the elderly the homeostatic responses become inadequate because of aging and of comorbidities and adverse effects may occur in situations that young people would cope with.

Frailty in gerontology defines a condition of general debilitation caused by several factors that should be taken into serious consideration because they can all contribute to an increased risk of an adverse drug reaction. Some of these multiple factors are hearing and vision disabilities confusion, balance and gait abnormalities, decreased knee strength, infrequent walking, all followed by loss of independence in daily activities and social deprivation. The number of dermoepidermal interdigitations and of subcutaneous fat decreases with aging, together with a loss of elasticity and a thinning of the collagen, with subsequent loss of the protective function of the skin. The number of medications used by individual patients probably represents the most relevant among all treatment induced risk factors. Patient adherence to therapy is an important determinant of adverse drug reaction occurrence together with pharmacokinetic interactions. Poor adherence of elderly to treatments are due to several factors, like living alone, depression, cognitive impairment, presence of psychological problems, complex treatment regimens, side effects, decision to omit doses, forgetfulness, representing many important barriers in the daily use of drugs [19].

## Gut

### Changes of aging in the gut

Changes in local immune responses of the digestive tract may contribute to the development of food allergies at any age. Like all other forms of allergy in the elderly, even food allergy appears to be increasing, although it may be masked by various symptoms corresponding with a general age-induced decline of physiological functions. Structural and functional changes or effects caused by drugs (e.g. acid-suppression medications), in addition to immunological alterations encountered at old age, might be responsible for food allergy development in the elderly [5]. Elderly people are at higher risk of food allergy due to their aging immune systems. The induction of mucosal tolerance has been proven to be compromised in elderly animals, while the effector phase is preserved. This may be reinforced by weakened secretory antigen-specific IgA responses and an increase in intestinal permeability with aging.

Another risk factor for older people in developing food allergies may also be the reduced digestive capacity of the stomach due to atrophic gastritis and/or antisecretive therapy. For example, proton pump inhibitors are among the most commonly prescribed drugs (40.9%) among italian people aged over 65 years [20]. In these contexts, undigested proteins can persist, overcome the epithelial barrier and stimulate cells of the immune system thus becoming allergenic. Indeed, mouse models indicate that these drugs support the induction of Th2 responses also in older animals and not only in young adults [21,22]. In recent year attention has been focused on the effects of aging on small bowel morphology and function. The active transport of certain substances is impaired in old age, although morphologic alterations do not appear to be responsible for this dysfunction.

Aging does not seem to be associated in humans with a reduction in the extent of the absorptive area. However, an increased proportion of relatively undifferentiated epithelial cells line the villi in the elderly, as suggested by the increased percentage of enterocytes expressing the cyclin proliferative cell nuclear antigen (PCNA) in elderly subjects, both at the level of villi and of crypts [23]. Therefore, the small intestine in old age is lined with enterocytes in the early phases of their maturation process. This relative immaturity of the aging gut does not have important morphological consequences, but may impair the absorptive function contributing to the epithelial dysregulation of the aged gut which predispose to allergy.

### Food allergy

Several reports suggest that food allergies are underdiagnosed among the elderly. However, looking at epidemiologic reports it appears that food allergy - while at large increasing in industrialized countries - does not occur in the elderly. This might be due to an underestimation, because these studies mainly focus on young adults and children, whereas the elderly population has not yet been adequately investigated in this regard. Therefore, food allergy is becoming a common health problem in old people. Innate and adaptive immune system are severely affected by age related changes and elderly patients are at higher risk of food allergy due to their aging immune systems. The development of allergies with aging, could also be sustained by micronutrients deficiencies, especially iron and zinc, as well as vitamin D, such as allergy can cause the deficiency of these elements [24,25,26].

A considerable increasing number of elderly patients present symptoms of food allergy. A study reported that in 24.8% of geriatric nursing home patients (mean age of 77) were detected specific IgE to food allergens [27].

The presentation of food allergy in the elderly can be different than pediatric-onset food allergy. Frequently late-onset food allergy occurs after exercise and consuming food to which the patients are allergic. Food allergy may present with a variety of symptoms involving cutaneous (e.g., urticaria, angioedema, eczema), respiratory (rhinitis, asthma), gastrointestinal (diarrhea), or generalized anaphylactic reactions [5].

The most frequent foods that elicit allergies in the elderly are fruits, vegetables, nuts, fish, shellfish. Sensitization to food allergens may occur directly or indirectly through cross-reactivity with aeroallergens. However, the ingestion of drugs is the most frequent cause of allergy in old people. Chronic alcohol consumption is among the risk factors for sensitization to food allergens in the elderly [27].

Eosinophilic esophagitis, an eosinophilic inflammation of the esophagus, represents another allergic manifestation more frequent in the elderly, with both immediate and late response to the ingested food [28].

## Airways

### Respiratory tract modifications

In the elderly several changes in the anatomy and physiology of both the upper and lower respiratory tract are observed.

At the level of the nasal mucosa, slower mucociliary transport time or increased dryness have been described. These age-related alterations may worsen nasal obstruction, itching, sneezing and rhinorrhea, which are typical symptoms of allergic rhinitis (AR) [29].

Nasal mucociliary clearance (NMC) clearly shows an increase with age signifying decreasing respiratory epithelium function. NMC is a primary innate defence mechanism of the nose and paranasal sinuses whereby mucus secreted into the upper airways by the goblet cells of the respiratory epithelium traps inhaled particulate matter, allergens, and pathogens and is then transported by the ciliated cells of the respiratory epithelium to the pharynx, where it is swallowed. Increased NMC time, signifying a decline in mucociliary clearance, could be attributed to a variety of anatomical, physiological, and biochemical changes which occur during the normal aging process, which may affect the response of the respiratory tract to inhaled agents. Impairment of NMC results in the accumulation of respiratory secretions and reduced lung defenses leading to infections and inflammation. Anatomical changes that occur with aging include nasal mucosal damage which has accumulated from infections over the years, ciliary ultrastructural defects such as the occurrence of increased central microtubular disorientation, altered proportions of elastic tissues and collagen and alterations in the cross-linkage of aging collagen. The impaired mucociliary clearance among the elderly is also the consequence of quantitative and qualitative changes in respiratory mucus and increased oxidative stress potential [30].

Changes in the tissues of the pharynx may lead to the leakage of food or fluids into the trachea during swallowing (aspiration). If persistent or severe, aspiration may cause pneumonia [31].

The geriatric larynx differs from the young adult larynx in many characteristic aspects: vocal fold bowing, prominence of the vocal process, glottic proportion, phase and amplitude symmetry of mucosal wave and tremor of laryngeal structures [32]. Scanning and transmission electron microscopic observations in the human laryngeal glands evidentiate the following findings: 1) granular endoplasmic reticulum and Golgi apparatus are sparse in the cytoplasm of serous and mucous cells, 2) secretory granules in serous cells are decreased in number, 3) secretory granules are less electron-dense compared to those in younger adult specimens, 4) mucigen droplets in mucous cells are diminished, and 5) discharge of secretory granules and mucigen droplets is decreased. These age-related morphologic changes in the laryngeal glands influence not only the amount but also the quality of secretions, leading to less lubrication of the vocal cords, thus causing aging of the voice. Tissue changes also include fatty infiltration of serous and mucous glands and fibrotic changes in connective tissue. Such age-related changes in the larynx partially alter also other laryngeal functions, including local immunity and mucociliary transport [33].

Moreover, aging results in the increased tracheal area and a distortion of the roundness, with consequent changes in air flow [34]. Epithelial cysts in the submucosal tissue underlying the lumen of the trachea and bronchi, in association with a decrease in the number and proportion of basal cells in the epithelium lining the airways, have been observed in aged mice. Global transcriptome analysis and flow cytometric data provide evidence for changes in gene expression and increase in the number of activated B and T cells in the aging trachea; these parameters are consistent with the development of low grade chronic inflammation. Therefore, senescence is associated with numerous changes in the cellular composition, organization and local microenvironment of the epithelium lining the upper airways [35].

In the lung, in the pathophysiology of asthma in the elderly, different biological processes associated with aging appear to be involved. Progressive loss of lung function in the course of the life and cell aging combined with destructive processes of inflammation represent some hallmarks of asthma in the elderly [36]. Elderly people have reduced defenses against infections, mainly pneumonia and influenza. Both the altered immune reactivity and the resulting infections causes local and systemic inflammation, changes in tissue airways and deterioration of lung function. The lungs lose more than 40% of their capacity (FRV) with aging. The age-related lung changes include airspace enlargement without alveolar destruction, loss of supporting tissue in the peripheral airways, and decreases in gas exchange surface with decreased arterial oxygen tension. The senescent lung is also characterized by a decrease of the static elastic recoil of the lung, and an increase of both residual volume and functional residual capacity. Moreover, in older people the chest wall compliance diminishes, the work of breathing increases, and the respiratory muscles become weaker. As a consequence of premature closing of distal airways, the expiratory flow rate decreases. Cough becomes less effective and thus unable to efficiently clear the lung. With ageing there is a decline in the sensitivity of the lung receptors and of respiratory centers leading to alterations in the perception of breathing associated to airway constriction. Sensitization to indoor allergens have a relevant role in determining asthma in the elderly. Moreover, since asthma is a chronic condition, there is an increased risk for developing new sensitizations. The airway epithelial barrier, formed by the apical junction of two adjacent ciliated cells (tight junctions), is less efficient in controlling the intercellular transport of inhaled particles in the elderly. The simultaneous decrease in immune regulatory cells contributes to determine inhalant allergen sensitization [37,38].

Immunosenescence and its associated chronic low grade systemic inflammation both have a driving role in the development and progression of lung diseases in the elderly, contributing to confer specific and unique clinical phenotypes to late-onset allergic asthma. The peculiar gut and airway microbiome have further impact on these phenotypes, implicating the interrelationship between host and microorganisms particularly in the setting of immunosenescence [39]. Immunosenescence, combined with lifelong impact of antimicrobial, allergic, and infective exposures, makes the microbiome of elderly people quite different from that of younger adults. These microbiomes associated airway variations mediate inflammation and subsequently confer age-related damaging airway changes [40].

ROS initiate the inflammatory response in the lungs by activating redox-sensitive transcription factors which promote the expression of pro-inflammatory cytokines such as tumor necrosis factor (TNF)-α, interleukin (IL)- 1, IL-6, and IL-8, subsequently inducing the activation of inflammatory cells in the airways. Aging is associated with elevated ROS levels. Age-related increases in ROS result in oxidative damage to intracellular components.

### Rhinitis and asthma

Rhinitis in its various forms can affect people of all ages and might significantly affect quality of life. In adults, it is recognized as a contributor to days off work, decreased productivity, and significant expense in additional health-care expenditures [41]. Although these data are not examined separately in older adults, it is expected that symptoms are similarly debilitating and costly. Nevertheless, rhinitis is often not adequately addressed in the health-care setting as it does not represent a life-threatening condition, and especially in the older population, there are often other health issues that take precedence. Etiologically rhinitis is divided into two types: allergic rhinitis (AR) and non allergic rhinitis (NAR).

AR is characterized by intermittent or persistent symptoms of nasal congestion, rhinorrhea, nasal/ocular pruritus, sneezing, and postnasal drainage. These symptoms are due to IgE-mediated allergic inflammation in the nasal mucosa. Several factors and inflammatory mediators drive the pathophysiology of allergic respiratory diseases in the elderly. Many of these mediators, also released in response to non-allergic triggers, enter the blood stream and sustain systemic inflammation, increasing the risk for cerebrovascular thrombotic events and other age-related chronic inflammatory diseases. Worsening of AR matches with asthma aggravation, whereas therapeutic control of nasal inflammation helps in controlling asthma. Allergic rhinitis in the elderly and exposure of the upper airways to inflammatory triggers such as pollutants, allergens and infectious agents, contribute to increase the risk of stroke and hospital admissions for other pathologies in aged people [38].

Rhinitis is one of the most frequent medical conditions. However, in older people, there is sparse epidemiologic evidence for rhinitis. Symptoms of rhinitis are common in elderly people, affecting approximately 32% of aged individuals in the US [42]. Nonallergic rhinitis, such as atrophic, infectious and vasomotor rhinitis, are very frequent among elderly. Thus an accurate differential diagnosis of any rhinitis should be carried out to provide the most appropriate management. The key element to the diagnosis of AR is demonstration of allergic sensitization to an allergen. This is typically done by either skin prick testing or serum testing for specific IgE to common seasonal (grasses, weeds, trees, molds) and perennial (house dust mite, pets, cockroaches) allergens. Although non allergic rhinitis increases with aging, approximately 33% of older adults were positive for inhalant allergen sensitivity on skin testing [43]. Photoaging is associated with a smaller response to histamine [44] and skin reactivity to diagnostic challenge with allergens, as well as serum IgE levels, are often decreased with aging, potentially impairing the diagnosis of allergy. Moreover, considering that the same age-related nasal changes predispose to allergic sensitization, allergic rhinitis in the elderly might be underdiagnosed.

Recently, Song et al. found a high prevalence of nonallergic rhinitis in the elderly, which was significantly related to asthma and quality of life [45]. Wuthrich et al. reported that in Switzerland in the population aged 60-70 years the prevalence of allergic rhinitis is of the order of 13-15% and should not be underestimated, even if in adults in adults aged between 18 and 60 years it is more frequent [7].

Positivity to nasal provocation test and detection of mucosal antigen-specific IgE secretion characterize local allergic rhinitis (LAR) in the absence of atopy by conventional measurements, i.e. skin prick test and specific serum IgE. There is no information about LAR in elderly people, just as there is little information about AR in this age group at large. Bozak et al. investigated the prevalence of LAR, AR, and non-AR in 219 elderly patients with rhinitis, with a mean (SD) age of 65.8 (5.9), nasal provocation tests, total serum specific-antigen IgE and skin prick tests against common aeroallergens. In addition, nasal specific IgE was measured in the nasal lavage at baseline and after provocation. The study showed that LAR and AR are common in elderly patients and that these conditions are often underdiagnosed [8]. A World Health Organization (WHO) report on active aging estimates that the proportion of individuals over the age of 65 years is expected to more than double by 2050. The burden of respiratory disease is concurrently supposed to increase in the same period, with already an estimated 300 million people worldwide suffering from asthma, with 250,000 annual deaths attributed to this disease [46].

There is little information on epidemiology of asthma and rhinitis in the elderly. Pite et al. in Portugal estimated in people older than 65 yrs. the prevalence of asthma, diagnosed by physicians and assessed according to allergic rhinitis and its impact on asthma (ARIA) recommendations. They also estimated the association of asthma with the presence and classification of rhinitis. This study showed that in aged people, asthma is a common disease and it has a strong association with rhinitis. According to ARIA classification, an especially high odds ratio for asthma is observed in persistent and severe types of allergic rhinitis [47].

Nowadays asthma represents a significant cause of morbidity and mortality in the elderly, while in the past it was regarded as a child and youth illness. Furthermore, the consequences of the diseases is more and more heavy in the elderly than in young especially with regard to mortality, hospitalization, health related quality of life and medical costs.

Frequently in old people asthma remains underdiagnosed (about 8% of diagnosis according to an epidemiological study in Portugal), and the percentage increases when respiratory symptoms are present [47]. Scarcely controlled asthma reduces the functional condition of older people, leads to a loss of independence, and to social withdrawal delaying the call for help and medical attention. Thus, it is essential to set future directions. Current knowledge suggests a phenotypical difference of asthma in older and young and this could potentially impact on diagnosis, assessment and management of the disease. The same diagnostic tests and clinical findings applied in young are used to diagnose asthma in the elderly, but interpreting clinical data becomes more difficult [48] Asthma in the elderly is broadly divided into patients with long-standing disease present from childhood, and late-onset disease describing those developing symptoms following the sixth decade of life. The diagnosis of the latter is particularly challenging as its symptoms are similar to alternative pathologies highly and increasingly prevalent in the older age-group such as chronic obstructive lung disease (COPD) or congestive cardiac failure (CHF).

Airway inflammatory cell types determine the physiological responses observed, and in the older asthmatic patients, eosinophilic inflammation is associated with airway hyperresponsiveness, while neutrophils are an important determinant of airflow limitation at rest and during bronchoconstriction. It should be noted that in the older asthmatic the exposure to chronic residential traffic pollution is associated with eosinophilic but not neutrophilic inflammation. These findings strengthen the relationship between airway pathophysiology and clinical relevant phenotypic differences observed in older patients with asthma [49]. Recent findings by Brandenberger et al. indicate the association of a Th17 response in old mice with enhanced allergic airway disease suggesting that elderly asthmatics could be more susceptible to develop severe allergic airway inflammation with a mixed Th2/Th17 immune response [50].

The inflammatory infiltration of the elderly asthmatic lung contains more apoptosis resistant inflammatory cells, which contribute to maintain persistent cell activation and worsen the disease. Both ROS damaged epithelia and infiltrated inflammatory cells produce growth factors which promote irreversible airway remodeling and ongoing inflammatory response. In addition to an increased prevalence of allergical determined respiratory diseases in the elderly, also mortality rates are highest among old asthmatic patients.

Although wheezing, dyspnoea, chest tightness, and other typical allergic asthma symptoms are also present in the elderly, the differential diagnosis with other age-related pathologies such as obstructive pulmonary disease, heart failure, gastroesophageal reflux, recurrent aspiration, pulmonary embolia, and tumors is potentially confounding. The allocation of medications impacting on respiratory functions should also be carefully considered. The progressive decline of pulmonary function in elderly patients with allergic asthma depends on age, duration of allergic disease, local and systemic comorbidities (metabolic syndrome, obesity, atherosclerosis, among others) and recurrent exacerbations triggered by repeated respiratory infections. The poorer prognosis and higher death rates of asthma in aged individuals also depend on the chronic systemic inflammation typical of senescence which underlies the frailty syndrome typical for aging [9]. Since aged people at least in industrialized countries spend the majority of their time indoors, in elderly patients those suffering from allergic rhinitis and asthma, the sensitization to indoor allergens, to whom they are mainly exposed, have a relevant role [51].

The severity of asthma strongly correlates with increased reactive oxygen species (ROS) levels which contribute to the airway inflammation. Thus, asthmatic symptoms related to oxidative stress in aged patients may be significantly affected by age-associated ROS. Additionally, oxidative stress and the resulting cellular modifications are prevalent in asthmatic patients during aging [52].

Asthma is a chronic airway inflammation with features of both heritability as well as environmental influences. Numerous asthma and allergy-related genes are susceptible to epigenetic control. Thus, the contribution of specific epigenetic regulation to allergic diseases in young and elderly people is a crucial topic [53]. Discovering and validating epigenetic biomarkers linking exposure to allergic diseases could help to a better definition of risk, prognosis, response to therapy and development of novel treatments also in elderly patients [54].

## Skin

### Dermatologic changes

Aging of the skin is characterized by loss of hydration and consequent dermis and epidermis atrophy and structural and functional integrity, with altered skin barrier function and reduced immunological response especially due to components of the extracellular matrix vascular impairment and the resulting metabolic disturbance and oxidative stress [55].

The barrier function depends on the stratum corneum but also on the epidermal tight junctions which play important role in maintaining the skin barrier properties. Although tight junctions have great self-regenerative potential and some barrier functions, such as transepidermal water control, are quite unchanged during aging, ultraviolet radiations, free oxygen radicals and photoaging, mainly due to exposure to UV radiation, impair the tight junction structure, skin barrier function and its recovery in the elderly, leading to facilitated allergen penetration into tissues [56].

A gradual reduction of almost all immune system functions accompanies senescence. In older adults, it has long been recognized that total IgE levels are lower compared to those in younger people. However, in the older adults with relatively higher levels of IgE, atopic disease can be found in a higher prevalence [57]. De Amici et al., to study the role of age on IgE production, assessed allergen-specific and total IgE production in a wide cohort of patients with allergies. The respective study included 6370 allergic patients and demonstrated a substantial variation in IgE production for different age groups. Furthermore, all antigen-specific IgE levels evaluated, showed differences in the various patients age groups, and each allergen had a specific trend pattern. Getting older the skin total production of IgE increases while specific-allergen IgE levels diminishes and each tested allergen show a specific trend [58].

### Allergic skin manifestations and adverse drug reactions

Among all adverse drug reactions, immunologically mediated (allergic) reactions represent a minority, but when a cutaneous drug reaction occurs more often, it is a drug induced allergic reaction. Many different lesions can be observed as the result of a drug induced cutaneous allergic reaction, and the recognition of the various skin patterns is fundamental for the diagnosis and the management of these allergic conditions. A mixed immunopathogenesis of some skin reactions (eg urticaria) may add confusion to the clinical picture. An extensive review and discussion of the different adverse drug reactions of the skin, has been done elsewhere [59], but some of the most common and severe among them will be pointed out below. Different clinical pictures of cutaneous adverse drug reactions in the elderly are described, varying from xerosis only or contact dermatitis (mostly due to topical agents) to urticaria, lichenoid rash, morbilliform rash, cutaneous vasculitis and drug induced autoimmune reactions, including lupus erythematosus, bullous pemphigoid and pemphigus related to the use of systemic agents. Topical sensitization is more frequent than oral route sensitization. Moreover, also drugs administered by injection and inhalation can cause skin reactions. When observing morbilliform, lichenoid or urticarial lesions with or without eczema, on the skin of older individuals with an itching rash, the most likely diagnosis is adverse reaction to systemic medication.

### Coutaneous type I allergies

The dryness of the skin favors the onset of both allergic contact and atopic dermatitis [60]. Ingestion or contact of potential allergens may result in allergic skin manifestations. It is necessary to differentiate itching/pruritus either derived from several causes like systemic and bullous diseases or xerosis or from allergic skin disorders. In developed countries the frequency of atopic dermatitis in older population is gradually increasing as the society ages, and recently new subtypes of atopic dermatitis were characterized and described in the elderly [61]. Atopic dermatitis in the elderly shows three major patterns of onset: a) as a first event in old age, b) as a relapse of the classic childhood form, c) as a relapse and/or continuation of adult disease. In old age atopic dermatitis, similar to other age groups, two forms are observed: the extrinsic or allergic IgE mediated and the intrinsic, non IgE mediated. House dust mites represent the most frequent environmental allergen involved in the extrinsic form, followed by pollens and food [62].

Atopic dermatitis in older people substantially reply the same pictures described in adults, even though the classic sign of localized lichenification is less frequent than the reverse sign of lichenified eczema around unaffected folds of the elbows and knees.

To date, the clinical aspects of atopic dermatitis of the elderly is well characterized but other relevant features remain to be clarified. Diagnosing atopic dermatitis in the elderly could be difficult because older people frequently suffer for several different itching cutaneous disorders as asteatotic dermatitis, chronic prurigo, adverse drug reactions, senile prurigo, and all these disorders have similar skin manifestations. For this reason, we need more objective and specific criteria to diagnose atopic dermatitis in the aging population, other than the standard clinical criteria, such as itching, typical skin lesions and multiple allergic sensitization. Further research should be conducted to understand the role of IgE mediated allergy in immunopathogenesis of atopic dermatitis of the elderly. In fact, IgE mediated inflammation is likely to play a key role in the pathogenesis of elderly atopic dermatitis as described in other age groups [63].

The elderly is frequently affected by xerosis, and the main cause is represented by the reduced activity of the sweat glands and sebaceous secretion. Even comorbid conditions and some medications, particularly antiandrogen therapies and diuretics, easily expose older people to develop this condition, so that in a nursing home it has been found that about 49% of the guests had xerosis [64].

### Cutaneous type IV allergies

Delayed-type hypersensitivity skin reactions (type IV) are mediated by inflammatory cytokines produced by infiltrating T lymphocytes and activated dendritic cells in the skin. Cytotoxic effector immune pathways are induced by topical haptens, that easily penetrate the dysfunctional aged skin barrier. Moreover, in the elderly the activation of negative regulatory pathways is also less efficient in down-regulating the inflammatory response [65] Symptoms of the acute phase are erythema, blisters and itching; if it persists, the vescicles are replaced by scaling, and the skin becomes very dry and itchy.

Nichel and balsam of Perù or fragrance represent the two allergens, most frequently detected with patch testing in the elderly, although several substances may be at the origin of contact dermatitis in older people. Also paraphenylenediamine, a substance present in dark hair dyes, is frequently reported positive. The long term application of topical medications used for venous stasis ulcers is associated with high incidence of multiple contact allergens.

The range of severity of skin drug eruptions goes from an asymptomatic rash to a life-threatening emergency, they have high frequency, morbidity and potential mortality. For these reasons it is important to be able to promptly recognize, work up, and treat patients with possible dermatologic drug reactions.

The geriatric population is at particular risk for drug eruptions. A study published 2006 by Yalcin et al. during a 5-year period studying 4099 geriatric patients found a prevalence of 1.4 % of cutaneous adverse drug reactions. It is unclear if the increased risk is due to polypharmacy alone or also to changes in drug metabolism and/or excretion with age [66].

As populations get older worldwide, paralleling the increased drug prescription in this age group, we observe a high prevalence in the elderly of adverse drug reactions. This group of people is at even greater risk for this condition due to polypharmacy, that is the assumption of five or more medications. More frequently skin adverse drug eruptions are mild but may be responsible for a poor quality of life in the elderly. The benefits of a therapy appropriately prescribed are indubitable, but to ensure a better quality of life to older people it is necessary to prevent and otherwise promptly recognize a possible adverse skin drug related reaction [67,68].

All stages of the passage of a medication through the body may change with aging and the relevance of these changes may significantly differ from individual to individual. The most relevant pharmacokinetic modification in older people is the reduction in renal drug elimination, mainly caused by the progressive development of kidney glomerulosclerosis.

Achlorhydria and hypochlorhydria may be the result of a reduced gastric acid secretion observed in the course of aging due to the dysfunction of gastric mucosal parietal cells. Thus, medication prescribed for the treatment of peptic ulcer disease or gastroesophageal reflux disease may result in higher concentrations and consequent potential adverse effects of other drugs, such as acid-labile antimicrobials as penicillin or erythromycin.

## Conclusions

As the population ages, clinicians must be prepared to meet the elderly's changing health care needs. To meet these patients' needs their specific and unique circumstances must be considered. Allergic diseases represent an old/new emerging health issue. Although atopic diseases most commonly present in the first three decades of life, many of these diseases may persist, and others may occur in old age de novo, respectively. Additionally, because many common illnesses mimic as atopic disease, the differential diagnosis of suspected allergic diseases becomes more expanded in an aging population.

Research in the field needs to focus on both human and animal model systems to investigate the impact of the aging process on the immunologic pathways underpinning allergy and its different facets. Epigenetic, environmental, and microbiological triggers need to be considered in the clinical setting, while translational investigation of novel therapeutic targets must be pursued.

Our current knowledge base is largely from studies in young adults, while as shown elderly people represent a different setting. Future work should not be limited to therapeutic targets, but also should identify new biomarkers of disease, accounting for the unique phenotypic makeup of the elderly allergic and for the immunological and physiological changes associated with the natural process of aging that affect diagnosis and management (Table 3).

**Table 3:** Future perspective

• New and detailed epidemiological research is needed. Most studies on allergy prevalence were conducted in childhood than in adulthood, when immune-senescence, concurrent diseases, polytherapy and adverse drug reactions could be frequent confounding factors and could potentially impact on diagnosis, assessment and management. • Research in the field needs to focus on both human and animal model systems to investigate the impact of the aging process on the immunologic pathways underpinning allergy and its different facets. • Particular attention must be paid to diagnostic tools. The same diagnostic tests and clinical findings applied in young are used to diagnose allergy in older people, but interpreting clinical data becomes more difficult in the elderly. Future research should also address the identification of possible new biomarkers for early diagnosis and prognosis in the elderly. While new standardization of old diagnostic procedure is essential in a geriatric setting. • The contribution of epigenetic regulation to allergic diseases is another crucial topic that needs to be further investigated. The identification of more specific biomarkers and key pathogenic molecules will add valuable insights into molecular networks operative in allergic diseases in the elderly. Moreover, it could be the base for both discovering potential mechanisms linking environment and epigenetics, and for modern therapeutic approaches. Indeed, new treatment strategies must be developed: translational investigation of novel therapeutic targets must be pursued.

This unstructured review of the literature could serve to stimulate new ideas and projects for the future identifying weaknesses in the fields of clinical practice and of research in geriatric allergology. So we could improve outcomes for older people suffering for allergic diseases proposing new directions for research and suggesting new therapeutic strategies. While encouraging new research, it is equally important apply our current knowledge to reach a timely and accurate diagnosis of allergy, to develop the best therapeutic approach to reduce the discomfort and to control the disease, to educate the patient, and ultimately to ensure a better quality of life to our aging patients thorough a deep translational vision of medicine.

In Tables 1, 2 and 3 the most significant aspects on the topic, open issues, and work to do are highlighted. Summarizing, the prevalence of allergic diseases in older population is large and increasing; the spectrum of allergic diseases is broad and the number of causative agents is ever increasing; allergens often cause symptoms in different organs simultaneously or sequentially and complicate an already compromised condition for several other cofactors; the social and economic impact of allergic diseases is considerable while old allergic patients are expected to appeal increasingly to novel diagnostic possibilities and healthcare facilities. For these reasons, it is necessary to better understand, in the elderly, the pathophysiologic mechanisms of allergy, also in the presence of comorbidities, of polymedication and of several other modifying circumstances, to update diagnostic and therapeutic guidelines, adherence to therapy and preventive measures.

Advances in our knowledge of biological mechanisms of allergic diseases and their risk factors, together with the increase in our understanding of the molecular and genetic basis of human senescence, are providing unprecedented opportunities for advances in the field of allergic diseases in the elderly. Such discoveries can be “translated” into new diagnostic and therapeutic applications. The interface between an epidemiological approach and basic and clinical research in this field, might provide the tools for a proper management of allergy in the elderly.

Furthermore, it is necessary to promote communication, share good practices and thus improve quality of care for old allergic patients. In particular, for a better and prompt recognition of allergic conditions in the elderly, it is necessary to improve interdisciplinary collaboration: general practitioners and geriatricians should be aware of this issue and ready to recognize several different conditions imputable to allergy, to promptly diagnose and manage also in cooperation with the allergist and/or the clinical immunologist.
